# Psychiatric genome-wide association study enrichment shows promise for future psychopharmaceutical discoveries

**DOI:** 10.1038/s43856-025-00877-9

**Published:** 2025-05-16

**Authors:** Alexander S. Hatoum, Aaron J. Gorelik, Lauren Blaydon, Spencer B. Huggett, Tingying Chi, David A. A. Baranger, Alex P. Miller, Emma C. Johnson, Arpana Agrawal, Ryan Bogdan

**Affiliations:** 1 Washington University in St. Louis, Department of Psychological & Brain Sciences, St. Louis, MO USA; 2 Washington University School of Medicine, Department of Psychiatry, St. Louis, MO USA; 3 Washington University School of Medicine, AI and Health Institute, St. Louis, MO USA; 4 Synapze LLC, Boston, MA USA; 5 St. Louis Behavioral Medicine Institute, St. Louis, WA USA; 6 Indiana University School of Medicine, Department of Psychiatry, Indianapolis, IN USA

**Keywords:** Psychiatric disorders, Genetic association study

## Abstract

**Background:**

Innovation in psychiatric therapeutics has stagnated on known mechanisms. Psychiatric genome-wide association studies (GWAS) have identified hundreds of genome-wide significant (GWS) loci that have rapidly advanced our understanding of disease etiology. However, whether these results can be leveraged to improve clinical treatment for specific psychiatric disorders remains poorly understood.

**Methods:**

In this proof-of-principal evaluation of GWAS clinical utility, we test whether the targets of drugs used to treat Attention Deficit Hyperactivity Disorder (ADHD), Bipolar Disorder (BiP), Generalized Anxiety Disorder (GAD), Major Depressive Disorder (MDD), Post-Traumatic Stress Disorder (PTSD), Schizophrenia (SCZ), Substance Use Disorders (SUDs), and insomnia (INS), are enriched for GWAS meta-analysis findings.

**Results:**

The genes coding for treatment targets of medications used to SCZ, BiP, MDD, and SUDs (but not ADHD, PTSD, GAD, or INSOM) are enriched for GWS loci identified in their respective GWAS (ORs: 2.78-27.63; all ps <1.15e-3). Enrichment is largely driven by the presence of a GWS locus or loci within a gene coding for a drug target (i.e., proximity matching). Broadly, additional annotation (i.e., functional: Combined Annotation Dependent Depletion [CADD] scores, regulomeDB scores, eQTL, chromatin loop, and gene region; statistical: effect size of genome-wide significant SNPs; Z-score of SNPs; number of drug targets implicated by GWAS), with the exception of weighting by the largest SNP effect size, does not further improve enrichment across disorders. Evaluation of prior smaller GWAS reveal that more recent larger GWAS improve enrichment.

**Conclusions:**

GWAS results may assist in the prioritization of medications for future psychopharmaceutical research.

## Plain Language Summary

To ensure the validity of genetic studies to discover new psychiatric treatments, we tested if large-scale genetic studies of psychiatric disorders could be used to rediscover existing psychiatric treatments. We found that by looking at results from genetic studies of schizophrenia, major depressive disorder, bipolar disorder, and substance use disorders we were able to rediscover existing treatments. The best indicator of the genes that would predict potential new medications was the degree to which the genetic change was associated with the original disease. Our findings should enable improved discovery of possible targets for psychiatric treatments.

## Abstract (web summary)

Hatoum et al. undertake exploratory analysis of genome-wide association studies of specific psychiatric disorders to find existing medications that treat those disorders and genetic pathways that may be used for future treatments. They find genes coding for treatment targets of medications and find effect size of the genetic marker to be a strong indicator of discovery potential.

## Introduction

Psychiatric disorders account for over 14% of deaths^1^ and 10% of lost healthy years globally^2^. Unfortunately, drug development to treat these disorders has stagnated^3^. Indeed, the gaps in our understanding of the etiology of psychopathology has predominantly restricted medication development to a limited number of known pharmacologic drug targets (e.g., dopamine receptor 2 antagonists, selective serotonin/norepinephrine reuptake inhibitors) that were often discovered by happenstance (e.g., treatments being developed to treat tuberculosis and pain were discovered to have antidepressant^4^ and antipsychotic properties^5^, respectively). More recent developments have focused on reducing side effects associated with these medications and broadening their targets (e.g., serotonin and norepinephrine reuptake inhibitors)^6^. As major treatment advances often directly target links within an etiologic chain (e.g., statins inhibiting cholesterol synthesis), leveraging our developing understanding of psychopathology to guide treatment development holds promise^6^. As a cost-effective starting point for the process of psychopharmaceutical discovery, recent large-scale psychiatric genome-wide association studies (GWAS) have reliably identified hundreds of common variants and pathways associated with risk^7–10^, that may be leveraged to prioritize drug targets for consideration in future drug repurposing studies as well as the development of treatments targeting novel mechanisms^11^. For example, early research across various categories of broad indications (neurological disease, cardiovascular disease, etc.) found that medications acting on pathways implicated by GWAS are more likely to be approved therapeutics for diseases of that indication and progress further in clinical trials^12^. More recently, GWAS have contributed to novel U.S. Food and Drug Administration^13^ (FDA) approved treatments for COVID-19^14^. Despite observations that genes implicated in GWAS (e.g., *DRD2*) code for proteins that are primary medication targets^8^, GWAS drug enrichment studies have yet to identify any currently approved treatments for psychiatric disorders^7,11,15,16^. For example, The largest studies of bipolar disorder (BiP) and major depressive disorder (MDD) GWAS have been found to be enriched for other medication classes but not for medication classes shown to treat these disorders (e.g., BiP: psycho-epileptics; calcium channel blockers^7^; MDD: estrogen targeting medications, and others^16^). For MDD, smaller studies have found inconsistent enrichment of antidepressants^17^. Further, when accounting for the number of targets of a treatment, there was no evidence of enrichment of current treatments for SCZ or BiP^11^. To date, drug enrichment studies of specific psychiatric GWAS have used gene-set analyses that test whether genes coding for medication targets are enriched for genome-wide significance (GWS) relative to all other genes or a random set of genes. Positive results emerge when medication targets are overrepresented among genome-wide significant findings relative to non-medication targets. While the gene-set approach has many strengths, including its implementation as a post-GWAS annotation tool and application as an agnostic approach, it may bias against rediscovering current treatments, making the validity of psychiatric GWAS difficult to evaluate. Indeed, the identification of non-therapeutic targets within a GWAS is irrelevant to the presence of signals from medication targets. In other words, for a GWAS to identify a current medication target, it does not matter if the GWAS also identifies other non-targets. As such, while testing for enrichment of genes coding for medication targets relative to other genes may provide valid data for GWAS, it does not directly facilitate the identification of treatment targets. Further, evaluating enrichment for targets of currently approved psychiatric medications relative to non-targets could bias results toward the null due to the limited number of possible medication targets and the existence of well-powered GWAS that have identified many loci, mostly within non-drug targets.

How medication targets and off-targets (proteins the drug binds to other than the intended target/s) are included and/or combined across medications in analyses may also bias the results of gene-set enrichment analyses. For example, large and heterogeneous medication target gene lists (e.g., combining across multiple classes of medication used to treat a given disorder or combining across treatments for multiple different disorders) may bias results toward positive associations due to chance alone. Indeed, unlike other studies that produced evidence of enrichment for non-typically prescribed medications^7,16^, the only study accounting for the number of medication target genes included produced null results for psychiatric disorders^11^. On the other hand, inclusion of therapeutically irrelevant off-targets could bias toward the null. Essentially, the inclusion of medication targets that are not responsible for the therapeutic effects of a drug and are not (or minimally) genetically associated with disorder risk would bias gene-set enrichment estimates downward. Overall, the currently used gene-set GWAS drug enrichment approach is qualitatively distinct from formally testing for the presence of medication that could be prioritized relative to others, and could contribute to the current lack of GWAS support for existing psychiatric treatments.

Here, we test whether currently approved medication targets are enriched for GWAS findings, a critical proof-of-principle that has yet to be established in psychiatry. We used a drug-set enrichment approach because the drug-set enrichment approach tests whether a medication or set of medications is enriched for GWAS findings relative to all other drugs (*n*_drugs total_ = 1201), as opposed to gene-set analyses, which test whether genetic variation within medication targets is more likely to be GWS than chance relative to all other genes(i.e., non-targets of the medication). We also chose drug-set enrichment because prior studies of broad medication classes have found evidence that drugs with support from GWAS significant SNP effects progress further through clinical trials^12^ and drug-set enrichment’s ability to compare existing medications, which would facilitate the prioritization of existing drugs that could be evaluated for psychiatric repurposing.

We applied drug-set enrichment to the following psychiatric disorders that had a GWAS with at least 3 independent GWS loci identified as well as specified treatments in Stahl’s Prescribers Guide^18^: (1) attention deficit hyperactivity disorder (ADHD, *n*_GWAS_ = 225,534^19^, *n*_treatments_ = 18), (2) bipolar disorder (BiP, *n*_GWAS_ = 413,466^7^, *n*_treatments_ = 25), (3) generalized anxiety disorder (GAD, *n*_GWAS_ = 241,541^20^, *n*_treatments_ = 35), (4) posttraumatic stress disorder (PTSD, *n*_GWAS_ = 174,659^21^, *n*_treatments_ = 25), (5) major depressive disorder (MDD, *n*_GWAS_ = 1,004,980^16^, *n*_treatments_ = 51), (6) schizophrenia (SCZ, *n*_GWAS_ = 320,404^8^, *n*_treatments_ = 31), (7) substance use disorders (SUDs, *n*_GWAS_ = 1,025,550^10,22^, *n*_treatments_ = 8), and (8) Insomnia (INSOM, *n*_GWAS_ = 386,533^23^, *n*_treatments_ = 15), a transdiagnostic feature of many psychiatric disorders that has dedicated treatments. In addition to evaluating enrichment of GWS loci within medications, we also evaluated whether additional GWAS and -omics data including statistical (effect size and *z*-score) and biological annotations (e.g., CADD score) may be leveraged to enhance enrichment estimates. We found that schizophrenia, bipolar disorder, major depressive disorder, and substance use disorders were enriched for rediscovering their current treatments. Enrichment was not improved by biological annotation, but was improved by the effect size of the marker SNP in the original GWAS.

## Methods

### Discovery GWAS

Summary statistics from the latest genome-wide association studies of psychiatric disorders were downloaded from publicly available locations, including the Psychiatric Genomics Consortium and dbGAP (see “Data availability statement”). We restricted to GWAS with at least 3 independent genome-wide significant loci. We included: (1) schizophrenia *N* = 320,404 (Wave 3; Trubetskoy^8^), (2) bipolar disorder (BiP) *N* = 413,466 (Wave 3; Mullins^7^), (3) major depressive disorder (MDD) *N* = 1,004,980^16^, (4) generalized anxiety disorder (GAD), *N* = 241,541^20^, (5) attention deficit hyperactivity disorder (ADHD) *N* = 225,534^19^, (6) posttraumatic stress disorder (PTSD) *N* = 174,659^21^, (7) general and specific substance use disorders, *N* = 1,025,550^10,22^. Insomnia (*N* = 386,533^23^) was also included as it is a transdiagnostic feature of many psychiatric disorders and has dedicated treatments, many of which (e.g., trazodone) are also used to treat psychiatric disorders. Eating disorders were excluded due to the lack of available treatments. Samples for negative control analyses using diabetes, psoriasis, epilepsy (negative control for all psychiatric disorders except BiP), and parkinson’s disease are described in the Supplemental Methods.

All data on this study is publicly and freely available to qualified researchers. All analyses used secondary data analysis, and no IRB was required. The MVP summary statistics for MDD, PTSD, and GAD were obtained via an approved dbGaP application (phs001672.v4.p1). For details on the MVP, see https://www.research.va.gov/mvp/. Data for schizophrenia, bipolar disorder, substance use disorders, MDD, bipolar disorder, GAD, PTSD, and ADHD were downloaded from the Psychiatric Genomics Consortium Website (see: https://pgc.unc.edu/for-researchers/download-results/) and is freely available for download to researchers without any commercial interest in their use (data use agreements apply). Diabetes data comes from DIAGRAM consortium website and is freely available for download: https://diagram-consortium.org/ GWAS summary statistics for epilepsy were downloaded from: https://www.epigad.org/, and are publicly available GWAS summary statistics for Psoriasis and Parkinson’s disease were downloaded from NHGRI-EBI GWAS Catalog at https://www.ebi.ac.uk/gwas/ and are publicly available.

### Drug-psychiatric disorder pairing

The *Prescriber’s Guide: Stahl’s Essential Psychopharmacology version 7* (Stahl’s guide)^18^, which comprehensively lists all psychopharmaceuticals (including off-label medications) used in the clinic to treat specific conditions, was used to identify drugs used to treat psychiatric disorders. To ensure that results were not specific to drug-disorder pairing derived from Stahl’s guide, we also tested alternative gene-drug coding schemes using opentargets.org and drug indication coding scheme using clinicaltrials.gov data (Supplemental Methods; Supplemental Table 6). As clinicaltrials.gov data was coded with failed clinical trials having a categorically lower value than successful ones, this analysis also tests whether GWAS data discriminate against clinical trial progression.

#### Defining drug targets

The Drug Gene Interaction Database (DGldb; which uses a dense literature search to assign drug targets^24^) and Connectivity Map (CMAP; which uses differential expression from human cell lines^25^; *N*_Drug_ = 1201^11^) were used to define drug targets and identify Gene–Drug pairings, as has been done previously (“Data availability statement”). Coding drug targets based on the OpenTargets.org database (Supplemental Methods) did not alter broad conclusions (“Results”).

### Drug-set enrichment analyses: disorder gene–drug pairing

Our drug-set enrichment analyses tested if drug targets (i.e., transcripts or proteins directly modulated by the drug) of therapeutics are enriched for GWAS signals associated with the psychiatric conditions they are used to treat. In other words, a drug–gene pairing was considered a positive enrichment case in our primary proximity matching analyses when a SNP reaching GWS in a GWAS of that disorder resides within a gene (±10 kbp) coding for a protein that is targeted by a therapeutic listed in Stahl’s guide as a treatment for that disorder (“Methods”).

Logistic regression models were implemented in base R (version 4.2.0)^26^ as follows:

$${\rm{Y}}={\beta }_{0}+{{\rm{X}}}_{1}{\beta }_{1}+{{\rm{X}}}_{2}{\beta }_{2}$$

Y = 0 or 1 if therapeutic treats the specific psychiatric disorder.

*β*_0_ = Model intercept.

X_1_ = Number of therapeutic drug targets (positive whole number integer) was included as a covariate. Because targeting the expression of many genes could lead to a therapeutic being more likely to be a treatment, and also more likely to contain a gene implicated by GWAS. In other words, our test is a competitive test compared to simply having a drug with more drug targets.

X_2_ = 0 or 1 based on whether any gene implicated by the GWAS is a target for the psychiatric disorder (i.e., 0 if no drug targets of that therapeutic were found in the GWAS, 1 if any drug target of that therapeutic is mapped to the GWAS). *β*_2_ is the primary parameter of interest, testing the null hypothesis of *β*_2_ = 0 against the alternative *β*_2_ $\ne$ 0, with a significant test indicating greater likelihood that a therapeutic with a drug target implicated by the GWAS is a current treatment used for that psychiatric disorder. As the outcomes are dichotomous values of overlap/no overlap, the test can be interpreted as odds increase in the chance that a drug paired with a disease by GWAS is a drug that is known to treat that psychiatric disorder, after accounting for the number of drug targets of each therapeutic (i.e., X_1_).

To test whether drugs with more drug targets implicated by GWAS were more enriched for current treatments, we also tested a model where X_2_ = a positive integer of the total number of drug targets implicated by the GWAS of that disorder. Sankey diagrams were used to visualize drug-gene pairings using the network3D package in R^27^. Analyses were repeated using permutation testing to ensure that findings were not driven by artifactual factors (Supplemental Methods).

Finally, we explored whether eQTL or conformational mapping, GWAS annotations (i.e., functional annotations including Combined Annotation Dependent Depletion [CADD] scores^28^, regulomeDB scores, and gene region [e.g., exonic, intron]), or other information (i.e., effect size of genome-wide significant SNPs; *Z*-score of SNPs; or number of drug targets of that therapeutic implicated by GWAS; GWAS sample size) potentiated drug target enrichment for GWAS signals beyond proximity matching with GWS loci (see Supplemental Methods).

### Reporting summary

Further information on research design is available in the Nature Portfolio Reporting Summary linked to this article.

## Results

**Fig. 1:**
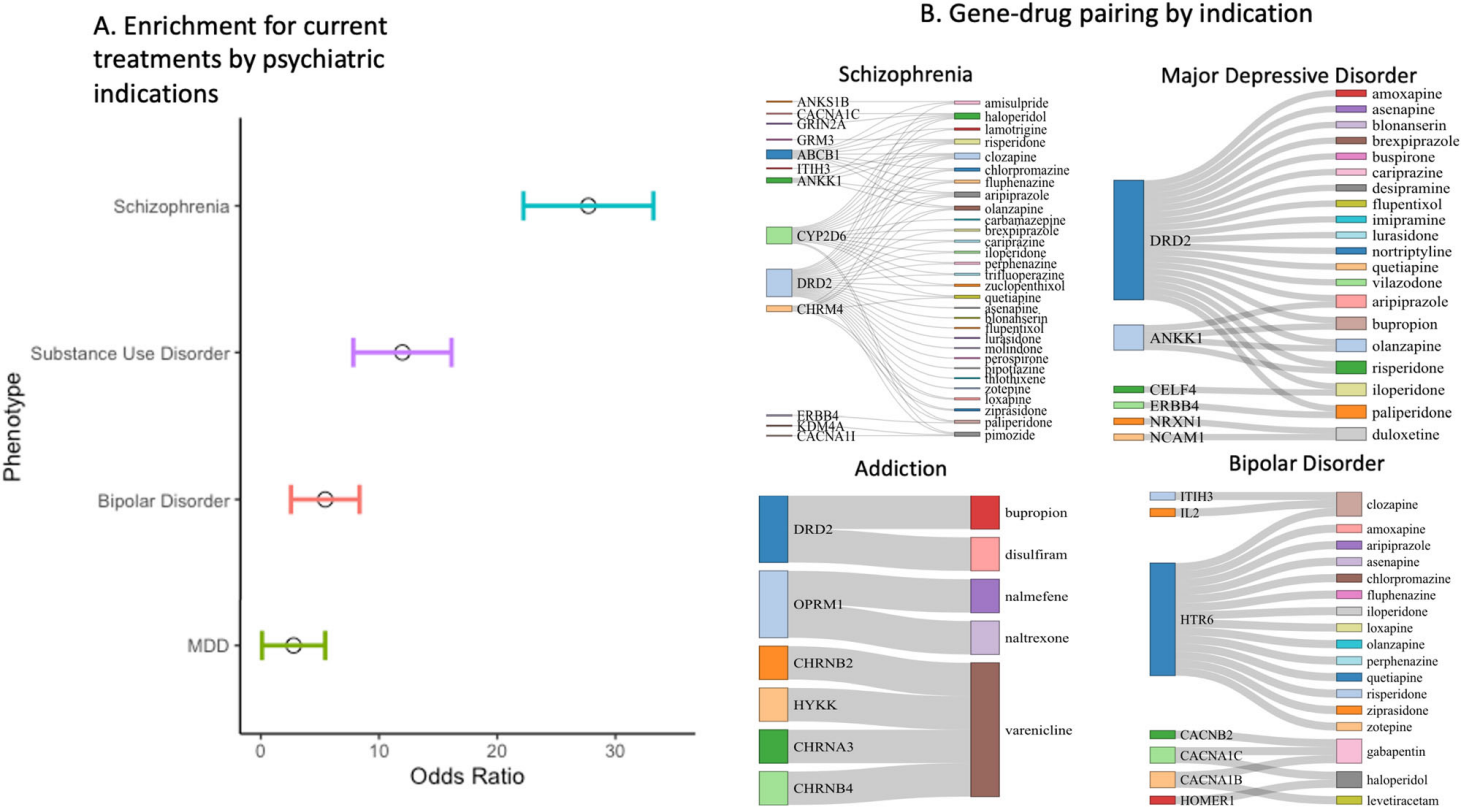
GWAS implicates current treatments for psychiatric disorders. **A** Odds ratio of a drug implicated by GWAS being a known treatment for a psychiatric disorder, controlling for the number of drug targets of each drug. Those results that were significant after Bonferroni are plotted with their standard error. MDD major depressive disorder, ADHD attention deficit hyperactivity disorder, GAD generalized anxiety disorder, PTSD post-traumatic stress disorder. **B** The link between a treatment for that disease and the drug targets from GWAS is shown as a Sankey Network Diagram. Larger colored edges signify genes (left) and drugs (right) that are more connected. These are the positive enrichment cases that are driving our significant findings.

Proximity mapping revealed that targets of medications used to treat SCZ, BiP, MDD, and SUDs, but not ADHD, PTSD, GAD, or INSOM, are enriched for GWS loci of their respective disorders, after Bonferroni correction for the number of disorders tested (odds ratios [ORs]: SCZ = 27.629; BiP = 5.398; MDD = 2.776; SUDs = OR = 11.95; all *p*s < 1.15e−3; Fig. 1A; Supplemental Table 1 and Supplemental Table 2; “Methods”). Gene–drug-disease pairings (total *n* = 74) included the following genes and medication classes, among others (Fig. 1B): (1) **SCZ**: dopamine (e.g., *DRD2 antagonists*-antipsychotics, clozapine), glutamate (e.g., *GRM3*, *GRIN2A*-NMDA receptor modulators, haloperidol and risperidone); (2) **BiP**: serotonin (e.g., *HTR6A*-antipsychotic class, quetiapine), and calcium channels (*CACNB2, CACNA1C, CACNA1B*-anticonvulsants, gabapentin); (3) **MDD**: Norepinephrine reuptake drugs (specifically duloxetine, and Nortriptyline, with *NRXN1* and *NCAM1 involved in modulation of chemical synaptic transmission*); (4) **SUDs**: dopamine (*DRD2*-dopamine agonists, bupropion), and substance-specific pathways (e.g. *CHRNB2-varenicline, OPRM1-naltrexone*). *DRD2* GWAS signals drove enrichment for all schizophrenia treatments; a *post hoc* analysis excluding therapeutics that were only enriched due to *DRD2* GWAS signals still revealed significant enrichment for SCZ treatments (OR = 19.49; *p* = 2.47e−4).

Permutation analyses of gene lists confirmed that findings remain significant beyond a random list of selected genes. More specifically, treatments for SCZ, BiP, and SUDs remained significantly enriched for GWAS findings (Supplemental Table 3, all *p* < 0.05) except MDD, which was significant in our primary analyses, was no longer significant. Further, our negative control analysis showed that psychiatric drug targets are not enriched for type 2 diabetes, psoriasis, or Parkinson’s disease GWS signals (*p* = 0.119–0.993; Supplemental Table 4). As anticipated, the epilepsy GWAS was enriched for treatments for bipolar disorder (*p* = 4.25e−6), consistent with anti-epileptic drugs being included as BiP treatments in Stahl’s guide, but not the other psychiatric disorders (*p* = 0.246–0.992).

We conducted a series of post hoc analyses to confirm the robustness of our results. First, we used alternative coding schemes for drug–gene pairing and drug-disease pairing OpenTargets.org and clinicaltrials.gov; Supplemental Methods). This analysis produced largely consistent results (all *p* = 0.002–3.07e−14; Supplemental Results, Supplemental Table 5). BiP was the exception, which was no longer significant (*p* = 0.645) when using opentargets.org coding, specifically. Second, as medications of similar class often have similar treatment effects and targets, analyses accounting for shared targets across therapeutics (Supplemental Methods) revealed that our results remained significant accounting for class (Supplemental Table 6).

Next, we tested whether drug targets are enriched for genes identified through local expression quantitative trait loci (cis-eQTL) and conformational (i.e., chromatin interactions: Hi–C) annotation of GWAS signals, and whether such signals may supplement proximity matching drug target enrichment for GWAS signals (Methods). eQTL or conformational mapping (i.e., Hi–C) alone revealed less consistent therapeutic enrichment than proximity mapping (Supplemental Table 7). When cis-eQTL and Hi–C annotations were used to supplement proximity mapping, there was evidence for drug target enrichment; however, enrichment values were nominally lower and within the confidence intervals of proximity mapping alone (Supplemental Table 8).

Finally, we explored whether additional GWAS annotations (i.e., functional annotations including combined annotation dependent depletion [CADD] scores, regulomeDB scores, and gene region [e.g., exonic, intron]), or other information (i.e., effect size of genome-wide significant SNPs; *Z*-score of SNPs; or number of drug targets of that therapeutic implicated by GWAS; GWAS sample size) potentiated drug target enrichment for GWAS signals (see “Methods”). When annotating SNPs with additional information, the only consistent increase in enrichment (as indicated by non-overlapping confidence intervals) across all disorders was the largest effect size of any SNP, which increased enrichment betas by 18- to 44-fold (Fig. 1 and Supplemental Table 9, Supplemental Table 10).

## Discussion

The multitude of mechanisms contributing to the expression of psychiatric disorders has made it challenging to identify biological pathways that can be therapeutically leveraged^6^. Indeed, recent treatment developments in psychiatry have been largely constrained to refinements of existing treatments that were developed decades ago^3^. By evaluating drug-set enrichment for psychiatric treatments, we show, for the first time, that medication targets of treatments for SCZ, BiP, MDD, and SUDs (Fig. 1), but not ADHD, PTSD, GAD, or INSOM, are enriched for GWAS findings of their respective disorders (Supplemental Table 1). Negative control analyses of other non-psychiatric disorders, permutation tests, modeling of shared treatment targets, and alternative medication coding schemes suggest that these findings are driven by GWAS signal as opposed to chance (e.g., number of gene targets or GWAS loci). There was little evidence that additional functional or statistical annotation further improved enrichment, with the exception of the maximum effect size across all SNPs on a gene–drug target. These initial proof-of-principle findings suggest that GWAS may usefully guide the prioritization of existing medications and establish an analytic pipeline that could be leveraged to potentially prioritize and guide drug repurposing evaluation.

Many drug targets with GWAS evidence (i.e., *ANKK1, CHRNB2*, *DRD2*, *OPRM1*) were the primary target of several existing medications (i.e., *DRD2* and *ANKK1* in bupropion, *OPRM1* for naltrexone, and *CHRNB2* for vernacline). For example, *DRD2* linked all antipsychotics to schizophrenia (Fig. 1); however, removing *DRD2* from the drug set still revealed significant enrichment for other schizophrenia medication targets, including off-targets. For example, the first generation antipsychotic DRD2 antagonist Haloperidol, was also linked to schizophrenia by the off-target *GRIN2A*, which encodes a subunit of the glutamatergic NMDA receptor involved in synaptic plasticity and learning (Fig. 1). This finding is consistent with evidence of glutamatergic disruption in schizophrenia that is associated with executive (dys)function^29–31^. Despite growing interest in targeting the glutamatergic system to treat schizophrenia, the utility of glutamatergic treatments for schizophrenia remains equivocal^30^. Other notable identified medication off-targets that included GWAS significant signal include genes (i.e., *NRXN1* and *NCAM1*) coding for cell-adhesion molecules involved in synaptic plasticity and neurodevelopment that are off-targets of the antidepressant SNRI duloxetine^25^. Additionally, the off-target of many bipolar disorder treatments, the serotonin receptor 6 (*HTR6*), as well as calcium channel targets (e.g., *CACNA1C*, *CACNA1B*), which align with prior gene-set enrichment findings^7^, were linked to psychopharmaceuticals via GWAS signals. That our pipeline revealed off-targets (e.g., glutamate, calcium channels) that have already received attention for medication development provides additional evidence for the validity and potential utility of our enrichment approach^32^.

Our proof-of-principle results show that the primary treatment targets as well as off-targets of current treatments for psychiatric disorders are enriched for GWAS signal, increasing confidence in the potential of GWAS data for future drug repurposing studies. As an example of how such data may be applied to identify existing treatments for other conditions that that could be repurposed for psychiatry, our prior multiomics analysis of SUDs identified *PDE4B*, a cyclic nucleotide phosphodiesterase involved in signal transduction, DA function, cognition, and neuronal plasticity^10,33^. While multiomics revealed 108 potential medications, *PDE4B* harbored a GWAS significant hit. Further the PDE4B inhibitor, Ibudialst, shows success in early clinical trials of opioids^33,34^, and alcohol^25^ use disorder. This suggests that GWAS and other sources of multi-omics data, including gene-set enrichment, may be used jointly to show evidence for new treatments. Indeed, past work has shown that considering gene-set analyses jointly with transcriptional analyses improves the discovery of existing treatments for MDD and schizophrenia^35,36^.

A few other findings warrant attention. First, our negative control analyses revealed that enrichment was specific to psychiatric disorders for which medications were intended to treat. Indeed, even more well-powered GWAS, such as those of type 2 diabetes, are no more enriched than chance for the psychopharmaceuticals that we examined. Our control analyses also found anti-epileptic medications were enriched for bipolar disorder as a positive control, but not all others, where it served as a negative control, in line with our coding scheme *from Stahl’s Prescriber Guide* (Supplemental Table 4).

Second, the majority of targets enriched for GWAS findings were unique to each disorder (e.g., *HTR6, CHRNB2, OPRM*1). However, 3 drug targets were shared by 2 or more disorders across disorders (i.e., *DRD2* for SCZ, MDD, and SUDs; *CACNA1C* for SCZ, BiP; *ANKK1* for SCZ and MDD; Fig. 1). As a large portion of the genomic architecture underlying psychiatric disorders is shared^37^ and psychotherapeutics for distinct disorders often target the same/similar pathways^3^, this is not surprising. It also highlights the potential utility of examining shared and unique genetic architectures associated with psychopathology.

Third, we found that existing pharmacological treatments for SCZ, BiP, MDD, and SUDs, but not ADHD, PTSD, GAD, or INSOM, were enriched for GWAS findings. Notably, the GWAS for disorders that yielded no evidence of medication target enrichment (i.e., ADHD, PTSD, GAD, and INSOM) are much smaller and identified fewer loci than those with enrichment evidence (Supplemental Table 1, Supplemental Fig. 1). This highlights the clinical importance of further expanding GWAS samples.

Finally, we find limited evidence that biological (i.e., CADD score, regulome score, and eQTLs or Hi-C chromatin contact) and statistical (i.e., ean and max *Z*-score of SNPs on genes whose expression is changed by the drug, mean and max effect size of the SNPs, and the number of genes implicated by the GWAS targeted by the drug) annotation further enhances enrichment beyond proximity matching. The one exception that led to improvement beyond proximity matching was weighting by the largest SNP effect size. As psychiatric disorders are highly polygenic disorders, one could hypothesize that integrating continuous information or information on multiple genes would improve enrichment. However, neither the *Z*-score nor the number of genes targeted improved enrichment. This lack of improvement may be attributable to the limited number of medication targets. Indeed, while psychiatric disorders are highly polygenic across the population^38^, the majority of current psychiatric medications have few targets, with psychopharmaceutical innovations historically focused on reducing the number of targets of a given drug^39^.

It is important to consider the limitations of our work. First, psychiatric disorders represent heterogeneous amalgamations of symptoms, with the same diagnosis having many distinct putative etiologies and comorbidity being commonplace^3^. Advances in nosology may facilitate a better understanding of shared and distinct etiologies to facilitate the isolation of more specific, and potentially less generalized, therapeutic pathways. Second, well-powered GWASs have largely been conducted with data from individuals whose genomes most closely resemble reference genomes of persons from Central Europe (or European-like), with limited but increasing representation from diverse global populations^40^. As sample size was shown by our study to be consequential in enrichment accuracy, concerted efforts to study diverse global populations are a priority. Third, there are large sex differences in psychopathology, yet GWASs may not inform sex-specific treatments as sex stratified analyses and evaluation of sex chromosomes are rare in psychiatric GWAS. Fourth, relying on GWASs for drug development prioritizes common variability in the genome; rare variants have been studied in schizophrenia and may cluster in similar areas of the genome as GWAS hits, but with larger effect sizes, suggesting potential for drug repurposing based on current analyses. Fifth, many loci associated with disorders through GWAS represent broad regulators of multiple pathways (e.g., *PDE4*) that may be difficult to target without inducing side effects. Finally and most critically, GWAS data alone cannot be used to determine pharmaceuticals that should be repurposed for psychiatric disorders. Preliminary in vivo models, quasi-experimental designs, and clinical trials are needed to truly repurpose compounds for clinical practice.

There are also limitations to our drug-set enrichment approach. First, in most instances, drug set enrichment can only consider SNPs above genome-wide significance. Genome-wide significance is an arbitrary threshold of association. In contrast, approaches like gene-set enrichment or the continuous *Z*-score and effect sizes approaches used here can consider SNPs beyond arbitrary significance. Second, the identification of drug targets is based on acute modifications of gene expression and proteins. However, many psychiatric therapeutics are known to not work until after weeks of treatment, potentially through secondary mechanisms (e.g., downstream effects of synaptic effects of cholesterol and TRKB-BDNF that may account for the temporally delayed therapeutic effects of some antidepressants)^41^.

Despite limitations, this proof of principle highlights the clinical potential of psychiatric GWASs to reify the promise of identifying novel treatment targets and repurposing existing drugs. Ultimately, the ability of GWAS to validate targets for psychopharmacological prioritization will improve innovation and reduce the disproportionate rising burden of mental illness.

**Fig. 2:**
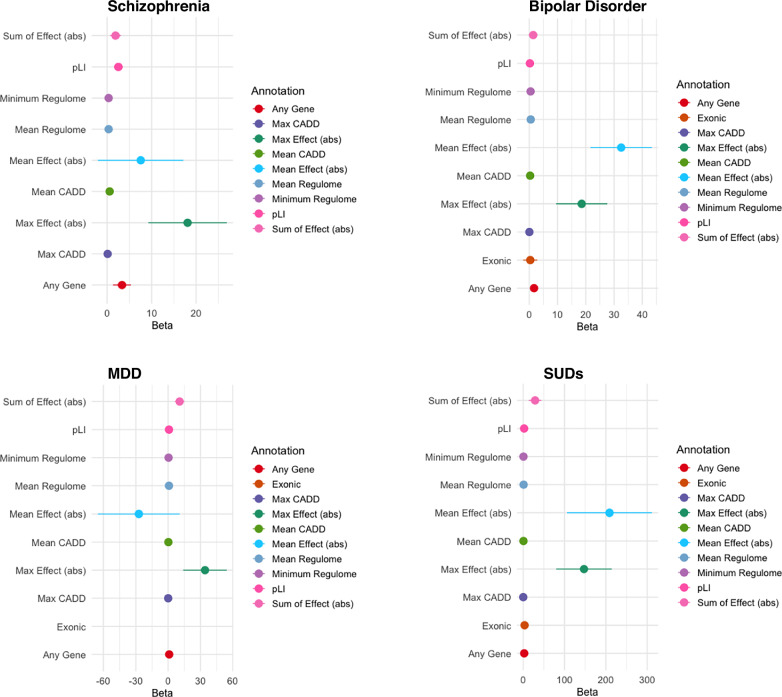
Enrichment for various genomic annotations. For each disorder we plot the enrichment when genomic annotations are used instead of 0 or 1 any-gene pairing. Each dot is the beta and CI’s for that beta. Abs absolute value or that the absolute value of SNP effect sizes was used, CADD combined annotation dependent depletion, pLI loss intolerance probability score. When points are missing from the plot, it is because no Exonic variant was a drug target after pruning SNPs for linkage disequilibrium. MDD major depressive disorder, SUD Substance use disorders.

## Supplementary information

Supplementary Material

REPORTING SUMMARY

## Data Availability

The MVP summary statistics for MDD, PTSD, and GAD can be obtained via a dbGaP application. Further details are at https://www.research.va.gov/mvp/ and in Gaziano et al. Million Veteran Program: A mega-biobank to study genetic influences on health and disease. J Clin Epidemiol 70, 214–223 (2016)^42^ to the reference list so you can add the citation number to the Data Availability statement. Data for schizophrenia, bipolar disorder, substance use disorders, MDD, bipolar disorder, GAD, PTSD, and ADHD can be downloaded from the Psychiatric Genomics Consortium Website (see: https://pgc.unc.edu/for-researchers/download-results/) and are freely available for download to researchers without any commercial interest in their use (data use agreements apply). Diabetes data from the DIAGRAM consortium are freely available for download at https://diagram-consortium.org/. GWAS summary statistics for epilepsy can be downloaded from: https://www.epigad.org/, and are publicly available. GWAS summary statistics for Psoriasis and Parkinson’s disease can be downloaded from NHGRI EBI GWAS Catalog at https://www.ebi.ac.uk/gwas/ and are publicly available. FUMA a publicly available and free to use software that supports analysis of GWAS summary statistics. All annotation databases on FUMA are free to use. Download result files generated from FUMA for Schizophrenia, bipolar disorder, MDD, and substance use disorders for each annotation can be found here https://wustl.app.box.com/folder/258363210203?s=c94gup91y63r30zonr1rt4dhvs6wj4e9. Data sufficient to recreate Figs. 1, 2 is contained in Supplemental Table 1.

## Code availability

Scripts for all analyses for enrichment analyses are available here https://github.com/PsychopharmGWAS.
