# Supplementary material for: Psychiatric genome-wide association study enrichment shows promise for future psychopharmaceutical discoveries
Source: Commun Med (Lond). 2025 May 16;5:176. doi: 10.1038/s43856-025-00877-9 (PMC12084526; doi:10.1038/s43856-025-00877-9)
Supplement: Supplementary file 1 — Supplementary Material [file 43856_2025_877_MOESM1_ESM.pdf]

Supplemental Materials

Index

Pg. 1 Supplemental Results
Pg. 14 Supplemental Methods

Supplemental Tables

Pg. 2 Supplemental Table 1:Enrichment Values By Each Disease Test for Treatments of That Disease
Pg. 3 Supplemental Table 2: Numbers of Overlapping Categories in Enrichment Analysis
Pg. 4 Supplemental Table 3: Enrichment Analysis with Significance Determined by Permutations
Pg. 5 Supplemental Table 4:Enrichment of Negative Control GWAS for Psychopharmaceuticals
Pg. 6 Supplemental Table 5: Enrichment in a mixed effects model with random intercepts for class or Super Class
Pg. 7 Supplemental Table 6: Enrichment for Significant Disease-Drug-Gene traits by Alternative Coding Schemes
Pg 8 Supplemental Table 7: Enrichment Using Cis-eQTL or HiC to Assign Mapped Genes Instead of Closest Gene
Pg. 9 Supplemental Table 8: Enrichment Supplementing Proximity Mapping with Cis-eQTL and Hi-C
Pg. 10 Supplemental Table 9: Enrichment of Psychopharmaceuticals by Various Genomic Annotations
Pg. 12 Supplemental Table 10. Enrichment When Drugs are Annotated by Continuous Z-score

Supplemental Figures

Pg. 13. Supplemental Figure 1: Sample Size Dependency of Enrichment

Supplemental Results

Alternative Coding Scheme Results. We reran enrichment analyses using two alternative coding schemes in order to evaluate robustness. As an alternative coding scheme to Stahl's prescriber guide to assign drug-disease pairing, we added information on clinical trial stage completed using clinicaltrials.gov. As an alternative to the gene-drug pairing using cMAP and Drug Interaction Database we used OpenTargets.org. Our results were largely consistent with the main results.

Using clinicaltrials.gov to assign alternative drug-disease indications yielded significant results for bipolar disorder, schizophrenia, and major depressive disorder **(Supplemental Table 4**). Substance use disorders could not be tested because the combined category was not possible as many drugs that work for one disorder are tested and failed for others at different clinical trial stages.

We used an alternative gene-drug pairing using OpenTargets.org. We found significant enrichment for Schizophrenia, Major Depressive disorder, and SUDs. We did not find significant enrichment for bipolar disorder. Upon closer inspection, for most psychiatric medications OpenTargets.org contains more restricted lists of gene-drug targets that are only the known functional target that is thought to produce the effect. This means that serotonergic targeting drugs only listed *SLC6A4* or *SLC6A2*, and typically just *SLC6A4*. This left out their known receptors, which drove many results in bipolar disorder in the main analysis (namely *HTR6A*). We concluded through exploration that these lists were likely overly restrictive. For example, Nortriptyline does not list *DRD2* or SNRI targeting mechanisms, but is a well-known modulator of norepinephrine and a suspected modulator of dopamine, in addition to the main mechanism of action. In general, Tricyclic antidepressant and anti-convulsants did not list off-targets, but these off-targets are often the mechanism of effectiveness for the "off-label" prescribed medications commonly used to treat bipolar disorder (for example *CACNA1C*). The assumption that we should limit to the main targets of the mechanism of a drug is also restrictive for future drug repurposing efforts, as off-targets may produce therapeutic effects that are desired. Notably, despite the conservative list, 3 of 4 traits remained significant using OpenTargets.org

GWAS discovery power is likely tied to enrichment. We conducted empirical analysis of the degree to which earlier smaller GWASs of SCZ, BiP, and MDD were enriched for psychopharmaceuticals, with smaller waves typically not enriched (**Supplemental Table S1**); 2) all GWAS with significant treatment enrichment identified more genome-wide significant loci than those that did not (i.e.,for MDD,Bip, SCZ mean N hits = 310 with a range of 86-656, vs N hits = 23 and a range of 4 to 77 for non-significant findings; **Supplemental Table 1** and **Supplemental Figure S1**), and 3) the number of discovered loci increases the odds of enrichment (**Supplemental Figure S1**; $r^2 = .6$ between enrichment effect size and independent significant hits found).

Supplemental Tables

Supplemental Table 1. Enrichment Values By Each Disease Test for Treatments of That Disease (Visualized in Figure 1)

Trait	Independent Hits	Mapped Genes	Log(Odds)	SE	P-value
**SCZ1**	12	77	-15.800799	799.240094	9.84E-01
**SCZ2**	352	452	3.704155	1.025073	0.000302
**SCZ3**	656	713	3.318882	1.026613	0.00123
**MDD1**	16	96	-12.271863	727.202703	0.986536
**MDD2**	295	303	1.02112	0.314147	0.00115
**BIP1**	10	8	-13.578407	771.499873	0.986
**BIP2**	23	64	0.030375	0.670221	0.964
**BIP3**	86	259	1.686041	0.394786	1.95E-05
**PTSD**	4	5	-13.181676	882.743585	0.988
**ADHD**	77	61	-14.837923	964.757768	0.988
**SUDs**	163	146	2.48076	0.75338	0.000992
**GAD**	7	6	-15.559783	787.28408	0.98423
**Insomnia**	35	25	-15.68601	1295.00539	0.9903

Note. Table of enrichment values across all traits studied. SE = standard error Schizophrenia 1 = Schizophrenia Wave 1, SCZ2 = Schizophrenia Wave 2, SCZ3 = Schizophrenia Wave 3 (main analysis), MDD1 = Major Depressive Disorder Wave 1, MDD2 = Major Depressive Disorder Wave 2 (main analysis), BiP 1 = Bipolar Disorder Wave 1, BIP 2= Bipolar Disorder Wave 2, BIP 3 Bipolar Disorder Wave 3 (main analysis), PTSD = Post-Traumatic Stress Disorder, ADHD = Attention Deficit Hyperactivity Disorder, GAD = Generalized Anxiety Disorder.

Supplemental Table 2. Numbers of Overlapping Categories in Enrichment Analysis

Trait	Linked GWAS genes	Known Treatments	Known Treatments Implicated
SCZ	13	31	30
MDD	6	51	20
BiP	7	25	17
SUDs	6	8	5

Note. MDD = Major Depressive Disorder, SCZ = schizophrenia, BiP = Bipolar disorder, SUDs = Substance use disorders

Supplemental Table 3. P-values for enrichment calculated from Permutation Analyses

**Trait**	**P**
Bip	0.05
SCZ	0.025
MDD	0.127
SUDs	0.011

Note. MDD = Major Depressive Disorder, SCZ = schizophrenia, BiP = Bipolar disorder, SUDs = Substance use disorders

Supplemental Table 4. Enrichment of a negative control GWAS for Psychopharmaceuticals by indication

**Trait**	**Beta**	**SE**	**P**
**Epilepsy**			
Scz	0.498	0.43	0.246
BiP	2.349	0.4129	4.25E-06
MDD	-0.194	1.033	0.851
SUDs	-13.659	1302.63	0.992
**Psorasis**			
Scz	-2.013	0.862	0.0197
BiP	-2.058	0.780118	0.00834
MDD	-2.026	0.767	0.00824
SUDs	-0.19879	1.246	0.873
**Parkinson's**			
Scz	-16.544	715.399	0.982
BiP	-2.33	1.212	0.0546
MDD	-1.905	1.092	0.0811
SUDs	-15.041	1324.424	0.991

Note. MDD = Major Depressive Disorder, SCZ = schizophrenia, BiP = Bipolar disorder, SUDs = Substance use disorders

Supplemental Table 5. Enrichment for Significant Disease-Drug-Gene traits by Alternative Coding Schemes

OpenTargets.org Enrichment	Beta	SE	P
MDD	2.088	0.37	1.71E-08
SCZ	3.041	0.4	3.07E-14
BIP	0.327	0.807	0.6448
SUDs	2.135	0.759	0.00488

ClinicalTrials.gov enrichment	Beta	SE	P
MDD	0.953	0.306	0.00182
SCZ	1.47	0.39	1.61E-04
BIP	1.12	0.362	0.00205

Note. Alternative coding scheme enrichment for opentargets.org to define drug-gene pairing and clinicalTrials.gov to define disease drug-pairing. SUDs could not be tested with clinicaltrials.gov since most clinical trials are for specific SUDs and restricted to single substances. Because of this, coding to resolve the degree of completed trial for a treatment would only be able to use information for a single substance use disorder trait at time. MDD = Major Depressive Disorder, SCZ = schizophrenia, BiP = Bipolar disorder.

Supplemental Table 6. Enrichment in a mixed effects model with random intercepts for class or Super Class

Trait	Beta	SE	P
**Class**			
**MDD**	1.0092	0.3807	0.00802
**SCZ**	2.68113	1.06402	1.17E-02
**BiP**	2.02914	0.56316	0.000314
**SUD**	2.479771	0.75342	0.000997
**Super Class**			
**MDD**	1.072497	0.3195	0.000789
**SCZ**	3.192711	1.030163	0.00194
**BiP**	1.54884	0.40171	0.000115
**SUD**	2.52565	0.75755	0.000856

Note. Enrichment values when modeling the enrichment as a logistic mixed effects model with a random intercept for class or Super Class as determined by ClassifyFire chemical analysis.

Supplemental Table 7. Enrichment Using Cis-eQTL or HiC to Assign Mapped Genes Instead of Closest Gene

**cis-eQTL enrichment**	**Beta**	**SE**	**P**
**MDD**	0.166	0.554	7.64E-01
**SCZ**	0.544	0.41	1.85E-01
**BiP**	1.686	0.395	1.95E-05
**SUDs**	0.503	0.594	0.397

**HiC Enrichment**	**Beta**	**SE**	**P**
**MDD**	1.441	0.313	4.26E-06
**SCZ**	4.058	0.741	4.28E-08
**BiP**	-0.319	0.689	0.643
**SUDs**	1.41	0.761	0.0642

Note. MDD = Major Depressive Disorder, SCZ = schizophrenia, BiP = Bipolar disorder.

Supplemental Table 8. Enrichment using any gene that mapped to eQTL, Hi-C or through proximity mapping

**Trait**	**Beta**	**SE**	**P**
**SCZ**	3.199	1.027	0.00185
**BiP**	1.431	0.409	0.000464
**MDD**	0.7801	0.315	0.0134
**SUDs**	1.286	0.311	3.48-e05

Note. MDD = Major Depressive Disorder, SCZ = schizophrenia, BiP = Bipolar disorder.

Supplemental Table 9. Enrichment of Psychopharmaceuticals by Various Genomic Annotations

	Schizophrenia			MDD		
	**Enrichment Beta**	**SE**	**P**	**Enrichment Beta**	**SE**	**P**
**Any Gene**	3.318	1.02	0.0012	1.021	0.3141	0.0012
**Sum of Effect (abs)**	1.882	0.6094	0.002	10.661	2.259	2.37E-06
**Max Effect (abs)**	18.081	4.5052	5.98E-05	34.263	10.324	0.0009
**Mean Effect (abs)**	7.533	4.902	0.124	-27.204	19.3161	0.159
**Max CADD**	0.113	0.027	3.12E-05	0.048	0.0152	0.0016
**Mean CADD**	0.566	0.156	0.000287	0.249	0.0899	0.0055
**Minimum Regulome**	0.346	0.0812	2.09E-05	0.4285	0.0816	1.51E-07
**Mean Regulome**	0.346	0.0812	2.09E-05	0.819	0.2468	0.0009
**Exonic**	-16.259	946.681	9.86E-01	NA	NA	NA
**pLI**	2.521	0.5143	9.49E-07	0.825	0.3774	0.0289
	**Bipolar Disorder**			**SUDs**		
**Any Gene**	1.686	0.3948	1.95E-05	2.48	0.7534	0.001
**Sum of Effect (abs)**	1.397	0.8042	8.23E-02	28.724	7.86	0.0003
**Max Effect (abs)**	18.546	4.633	6.26E-05	147.07633	34.3351	1.85E-05
**Mean Effect (abs)**	32.518	5.553	4.76E-09	208.786	52.4009	6.77E-05
**Max CADD**	0.046	0.0231	4.52E-02	0.1356	0.03633	0.0002
**Mean CADD**	0.301	0.1079	5.30E-03	0.6614	0.2079	0.0015
**Minimum Regulome**	0.47	0.0774	1.21E-09	0.6894	0.169	4.51E-05
**Mean Regulome**	0.522	0.0812	1.33E-10	1.055	0.263	6.04E-05
**Exonic**	0.3599	1.269	0.777	3.37	0.7781	1.52E-05
**pLI**	0.2255	0.7066	7.50E-01	2.22	1.0374	3.24E-02

Note. Enrichment for targets of psychopharmaceuticals when using genomic annotations and values of genomic annotations. pLI = protein truncating loci (0, 1), abs = absolute value, CADD = Combined Annotation Dependent Depletion. Visuzlied in Supplemental Figure 2.

Supplemental Table 10. Enrichment When Drugs are Annotated by Continuous Z-score

**Max**	**Beta**	**SE**	**P**
**Scz**	0.103	0.072506	0.15617
**BiP**	0.305	0.17706	0.0851
**MDD**	0.54213	0.146	0.00021
**SUDs**	0.7566	0.44	0.0857
**Mean**			
**SCZ**	-1.317	1.233	0.286
**BiP**	-2.47187	1.40202	0.0779
**MDD**	-0.5894	1.868	0.75231
**SUDs**	1.210566	1.76	0.492

Note. MDD = Major Depressive Disorder, SCZ = schizophrenia, BiP = Bipolar disorder, SUDs = Substance use disorders

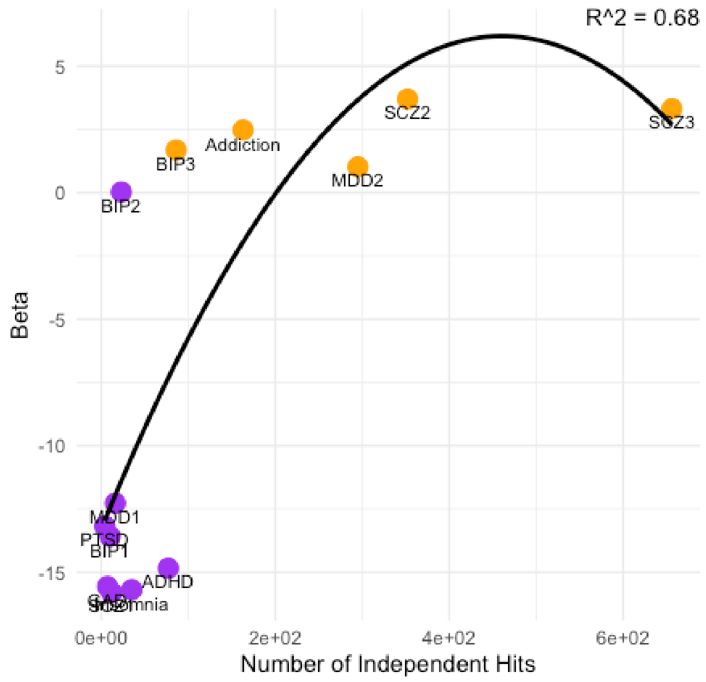

Supplemental Figure 1. Effect size of enrichment plotted against number of independent loci shows that enrichment of GWAS for current treatments is highly associated with the number of independent loci discovered by that GWAS. Beta representing an enrichment value is plotted on the y axis against the number of independent hits (SNPs with p-values less than 5e-8 that are greater than r^2 = .68 distance from each other). Each trait studied is noted by a single dot and a quadratic line is fit to the effect. Those traits that were significant are colored orange, those that are non-significant are colored purple. The figure shows there is a large and apparent effect of the number of GWAS independence loci discovered on enrichment of that GWAS for current treatments for that disorder. MDD2 = Major depression disorder wave 2, MDD1 = Major depression Disorder Wave 1, BIP1 = Bipolar Disorder Wave 1, BIP2 = Bipolar Disorder Wave 2, BIP3 = Bipolar Disorder Wave 3, SCZ 1 = Schizophrenia Wave 1, SCZ2 = Schizophrenia Wave 2, SCZ3 = Schizophrenia Wave 3, ADHD = Attention deficit hyperactivity disorder, GAD = generalized anxiety disorder, PTSD = Post-Traumatic Stress Disorder. Insomnia and SUDs are also shown.

Supplemental Methods

Negative Control Analysis. To test whether our results are specific to psychiatric disorders, or if any set of genome-wide associated genes would be associated, we conducted negative control analyses by testing GWAS of 3 other diseases for enrichment for medication targets. For this analysis, we chose diabetes (N = 1,339,889; Mahajan et al., 2022[1]) because it had more mapped genes than any psychiatric disorder, Psoriasis (N=44,161; Stuart et al. 2022[2]), because of the strongly associated gabaergic gene peak which is a neurologically expressed gene cluster that was more significant than any psychiatric disorders, and epilepsy (N= 69,995; International League Against Epilepsy Consortium on Complex Epilepsies 2023[3]) as inclusion of epilepsy could also act as a positive control for bipolar disorder where antiepileptics were included as treatments for bipolar disorder in our coding scheme. We also included Parkinson's disease (N = 200,684; Nalls et al. 2019[4]) as a negative control to act as a neurological disorder negative control, in comparison to epilepsy.

To enable tests of whether GWAS power/sample size impacts drug identification, we also used earlier GWAS waves of these same psychiatric disorders, which included: **1)** schizophrenia Psychiatric Genomics Consortium (PGC) wave 1 (N =51,695)[5], **2)** schizophrenia PGC wave 2 (N = 150,064)[6], **3)** bipolar disorder wave 1 (N = 16,731)[7], **4)** bipolar disorder wave 2 (N = 51,710)[8], and **5)** major depressive disorder (N= 480,359)[9].

SNP-Gene Pairing. ***Proximity Matching.*** FUMA[10] was used to assign independent genome-wide significant SNPs identified in GWASs to genes. FUMA is an online platform that facilitated analyses biological annotations. FUMA contains automated analyses of HI-C, CADD, and regulome scoring. FUMA also contains the encode datasets. FUMA is free and publicly available

when users create a sign-in and password. All databases FUMA uses are also publicly available through the platform. More information can be obtained on the website: https://fuma.ctglab.nl/. SNPs were first pruned based on $r^2 < .6$. The remaining loci were mapped based on physical location of being ± 10 kbp within a gene, excluding the MHC region due to its complex long range linkage disequilibrium. All GWASs were aligned to human genome build 37.

Drug-Psychiatric Disorder Pairing. The *Prescriber's Guide: Stahl's Essential Psychopharmacology version 7* (Stahl’s guide)[11] was used to identify drugs to treat ADHD (n treatments=18), MDD/depression (n treatments=51), schizophrenia/psychosis (n=31), bipolar/mania/maintenance (n treatments=25), PTSD (n treatments=25), GAD (n treatments=35), and insomnia (n treatments=15). Due to the small number of treatments per substance use disorder and the shared genetic etiology[12], substance use disorders were combined into a single category (alcohol use disorder/dependence, opioid use disorder, tobacco/nicotine dependence, cannabis use disorder; n treatments=8). This also allowed us to match to the largest study of generalized genetic risk for SUDs to date (Hatoum et al. 2023[12]). We chose Stahl’s guide for our primary analysis because the guide includes a combination of FDA approved therapeutics, along with commonly used therapeutics that are prescribed off label, and adjuncts. This gives us a more dense list of drugs that are likely to be true positives than relying only on the U.S. Food and Drug Administration approval. Double data entry was used to ensure accuracy of the coding scheme.

Alternative Coding Schemes. To test for robustness across coding schemes for drug-disease and gene-drug pairing, we considered an alternative gene-drug pairing coding scheme using OpenTargets.org[13] and an alternative drug-disease pairing scheme using clinicaltrials.gov. Open Target Genetics is an online tool and database which integrates functional and biological information from numerous sources including the literature and other large open science studies

(i.e., UK-Biobank) to enable identification of potential drug targets. For each drug we extracted a complete list of drug targets and their proximal genes. We labeled a drug as missing if the drug either did not have any potential drug targets or was not listed in the database. We used double data entry to ensure accuracy of this coding scheme.

As an alternative dataset of drug-disease pairing, we used clinicaltrials.gov, an open-source repository of clinical research studies (i.e., clinical trials and observational studies). Importantly, this database is reliant on both sponsors and/or investigators to submit records of their studies and only a limited review by the national library of medicine is conducted. The following inclusion criteria were used: 1) clinical trials; 2) interventional studies; and 3) trials phase (one through four). Trials with results and the following information were extracted from each drug for the highest clinical trial phase of each psychiatric disorder: 1) number of highest trial phase completed, 2) clinical trial status (e.g. if terminated, why); 3) type of result (i.e.., positive or negative); and 4) any additional notes to be reviewed by the rest of the coding team. We had a 4-level coding scheme for drug-disease pairing evidence. The highest degree of clinical trial success that is currently known (i.e., 1, 2, 3, 4), or if the drug is FDA approved but not on clinicaltrials.gov, it was given a 4. If it was in Stahl’s but not on ClinicalTrials.gov, it was given a 3. The variable was treated as a 4-level variable in a logistic regression to test for enrichment for each respective drug (see model below). This was to mirror past analyses that compared enrichment across stages of clinical development (see Nelson et al., 2015[14]). We used double data entry to ensure accuracy of this coding scheme.

Alternatives to proximity mapping: eQTL and Hi-C. Next, as an alternative coding scheme to genes in proximity, we remapped genes with two additional criteria. Specifically, we tested *cis*-eQTL and HiC chromatin loops to see if these biological characterizations improved enrichment

on their own, or when combined with proximity matching. Both used PsychENCODE[15] data to assign SNP annotations, with Hi-C using the PsychENCODE EP links and PsychENCODE Promotor Anchored loops specifically. eQTLs were considered significant at fdr corrected $p < .05$ in PsychENCODE.

Permutation Analysis. To test whether our gene lists were more associated than a random list of genes of equal length, we conducted a permutation analysis. In each permutation, we randomly selected genes at random until we had a list in equal length to the length of the list of genes mapped from GWAS. We conducted 10,000 permutations and an empirical distribution of their enrichment beta was generated. The analysis was run separately for each trait that was significant in the original analysis. Non-significant traits were not run. The beta value from our original analysis was then compared to the empirical null beta and compared for significance.

Accounting for Drug Class. Drugs in the same or similar drug classes will have a more similar molecular shape, and also are more likely to target the same condition. This means that drugs may not be statistically independent of one another. To account for this we took a drug class from ClassyFire[16] as a random factor that influenced drug clustering. Using lme4[17], we parameterized a random effects model with ClassyFire class as a random effect.

Functional Annotation and Effect Size to Improve Enrichment. We added information based on annotation categories and effect size of SNPs in the GWAS to see if this information increased drug enrichment for genes associated with the disorder through GWAS. Each category was tested independently for enrichment. Functional Gene Annotation was done using the SNP2Gene function on FUMA (see: https://fuma.ctglab.nl/tutorial#snp2gene10). Specifically, we examined whether the variant that implicated the gene paired to the therapeutic was exonic based on ANNOVAR[18] and whether the variant that implicated the gene was a protein truncating loci (pLI)

based on the Exome Aggregation Consortium (ExAC) database[19]. Next, we developed several measures by using existing SNP functional annotation scores. First, we used the Combined Annotation Dependent Depletion (CADD) score[20], a score that captures the expected deleteriousness of a SNP. We annotated therapeutics for the maximum CADD score of any SNP on the drug target for that therapeutic and the average CADD scores of all SNPs on drug targets for that therapeutic. We also used regulomeDB scoring[21], which gives a rank to non-coding variants based on their likely regulatory role, with lower values showing increased likelihood of a regulatory effect. We annotated therapeutics for minimum regulomeDB score number and average regulomeDB score.

Finally, we annotated therapeutics with several values representing the effect size of SNPs on the drug target. This included the mean Z-score of all SNPs on gene-drug targets for a drug (regardless of significance), max Z-score of all SNPs on gene targets for a drug, highest effect size of a SNP on any gene that is a drug target for that therapeutic, mean effect size of a SNPs on drug targets for therapeutic, and sum of the absolute value of effect sizes of SNPs on the drug targets for that therapeutic.

For each therapeutic we specified a similar model as our primary analysis, however, $\beta 2$ = the value for that annotation. e.g. for maximum CADD score, the value of $\beta 2$ = highest CADD score on DRD2 for most anti-psychotics. $\beta 2$ is tested against the null hypothesis of $\beta 2 = 0$ against the alternative $\beta 2$ 0 with a significant test indicating greater likelihood that a therapeutic with a drug target implicated by the GWAS is a current treatment used for that psychiatric disorder based on the value of that annotation. In this way, we can also include enrichment for continuous annotation categories. The model is specified as:

$$Y = \beta 0 + X1\beta 1 + X2\beta 2$$

Y = value for that annotation

$\beta 0$ = Model intercept

X1 = Number of therapeutic drug targets (positive whole number integer) was included as a covariate.

Method references

1. Mahajan, A. *et al.* Multi-ancestry genetic study of type 2 diabetes highlights the power of diverse populations for discovery and translation. *Nat. Genet.* **54**, 560–572 (2022).
2. Stuart, P. E. *et al.* Transethnic analysis of psoriasis susceptibility in South Asians and Europeans enhances fine mapping in the MHC and genome wide. *Hum. Genet. Genomics Adv.* **3**, 100069 (2021).
3. GWAS meta-analysis of over 29,000 people with epilepsy identifies 26 risk loci and subtype-specific genetic architecture | Nature Genetics. https://www.nature.com/articles/s41588-023-01485-w.
4. Nalls, M. A. *et al.* Identification of novel risk loci, causal insights, and heritable risk for Parkinson's disease: a meta-analysis of genome-wide association studies. *Lancet Neurol.* **18**, 1091–1102 (2019).
5. Schizophrenia Psychiatric Genome-Wide Association Study (GWAS) Consortium. Genome-wide association study identifies five new schizophrenia loci. *Nat. Genet.* **43**, 969–976 (2011).
6. Schizophrenia Working Group of the Psychiatric Genomics Consortium, S. W. G. of the P. G. *et al.* Biological insights from 108 schizophrenia-associated genetic loci. *Nature* **511**, 421–7 (2014).
7. Psychiatric GWAS Consortium Bipolar Disorder Working Group. Large-scale genome-wide association analysis of bipolar disorder identifies a new susceptibility locus near ODZ4. *Nat. Genet.* **43**, 977–983 (2011).
8. Stahl, E. A. *et al.* Genome-wide association study identifies 30 loci associated with bipolar disorder. *Nat. Genet.* **51**, 793–803 (2019).
9. Howard, D. M. *et al.* Genome-wide meta-analysis of depression identifies 102 independent variants and highlights the importance of the prefrontal brain regions. *Nat. Neurosci.* **22**, 343–352 (2019).

10. Watanabe, K., Taskesen, E., van Bochoven, A. & Posthuma, D. Functional mapping and annotation of genetic associations with FUMA. *Nat. Commun.* **8**, 1826 (2017).
11. Stahl, Stephen, M. *Stahl's Essential Psychopharmacology Prescriber's Guide*. (Cambridge University Press).
12. Hatoum, A. S. *et al.* Multivariate genome-wide association meta-analysis of over 1 million subjects identifies loci underlying multiple substance use disorders. *Nat. Ment. Health* **1**, 210–223 (2023).
13. Open Targets: a platform for therapeutic target identification and validation | Nucleic Acids Research | Oxford Academic. https://academic.oup.com/nar/article/45/D1/D985/2605745?login=false.
14. Nelson, M. R. *et al.* The support of human genetic evidence for approved drug indications. *Nat. Genet.* **47**, 856–860 (2015).
15. PsychENCODE Consortium *et al.* The PsychENCODE project. *Nat. Neurosci.* **18**, 1707–1712 (2015).
16. ClassyFire: automated chemical classification with a comprehensive, computable taxonomy | Journal of Cheminformatics | Full Text. https://jcheminf.biomedcentral.com/articles/10.1186/s13321-016-0174-y.
17. Bates, D., Mächler, M., Bolker, B. M. & Walker, S. C. Fitting linear mixed-effects models using lme4. *J. Stat. Softw.* **67**, 1–48 (2015).
18. Wang, K., Li, M. & Hakonarson, H. ANNOVAR: functional annotation of genetic variants from high-throughput sequencing data. *Nucleic Acids Res.* **38**, e164 (2010).
19. Karczewski, K. J. *et al.* The ExAC browser: displaying reference data information from over 60 000 exomes. *Nucleic Acids Res.* **45**, D840–D845 (2017).
20. Rentzsch, P., Witten, D., Cooper, G. M., Shendure, J. & Kircher, M. CADD: Predicting the deleteriousness of variants throughout the human genome. *Nucleic Acids Res.* **47**, D886–D894 (2019).

21. Dong, S. & Boyle, A. P. Predicting functional variants in enhancer and promoter elements using RegulomeDB. *Hum. Mutat.* **40**, 1292–1298 (2019).
